# Variations in physical activity and sedentary behavior during and after hospitalization in acutely admitted older medical patients: a longitudinal study

**DOI:** 10.1186/s12877-022-02917-8

**Published:** 2022-03-15

**Authors:** Baker Nawfal Jawad, Janne Petersen, Ove Andersen, Mette Merete Pedersen

**Affiliations:** 1 Department of Clinical Research, Copenhagen University Hospital Amager and Hvidovre, Copenhagen, Denmark; 2 The Emergency Department, Copenhagen University Hospital, Amager and Hvidovre, Copenhagen, Denmark; 3 Department of Clinical Medicine, University of Copenhagen, Nørre Allé 20, DK-2200 Copenhagen-N, Denmark; 4 Center for Clinical Research and Prevention, Copenhagen University Hospital, Bispebjerg and Frederiksberg, Copenhagen, Denmark; 5 Section of Biostatistics, Department of Public Health, University of Copenhagen, Copenhagen, Denmark

**Keywords:** Accelerometer, physical activity, sedentary, hospitalization, post-acute care, older adults

## Abstract

**Background:**

Inactivity is frequent among older patients during hospitalization. It is unknown how patients' daily activity pattern (diurnal profile) vary between hospitalization and after discharge. This study aims to describe and compare the distribution of physical activity and sedentary behavior in acutely hospitalized older patients during hospitalization and after discharge.

**Methods:**

We included data on 80 patients (+65 years) admitted with acute medical illness from the STAND-Cph trial. Physical activity and sedentary behavior were measured as daily number of steps, uptime (walking/standing) and sedentary behavior (lying/sitting) with an activity monitor (activPAL3, PAL Technologies Ltd). The patients wore the monitor for three periods of one week: during hospitalization, after discharge, and four weeks after discharge.

**Results:**

The patients’ median age was 80 years [IQR: 75;88], 68% were female and the median De Morton Mobility Index (DEMMI) was 57 [IQR: 48;67]. The daily median uptime was 1.7 h [IQR: 1;2.8] during hospitalization, 4.0 h [IQR: 2.7;5.4] after discharge and 4.0 h [IQR: 2.8;5.8] four weeks after discharge. The daily median number of steps was 728 [IQR: 176;2089], 2207 [IQR: 1433;3148], and 2622 [IQR: 1714;3865], respectively, and median daily sedentary behavior was 21.4 h (IQR: 20.7;22.4), 19.5 h (IQR: 18.1;21.0) and 19.6 h (IQR: 18.0;20.8), respectively. During hospitalization, a small activity peak was observed between 9-11 AM without any notable variation after. At discharge and four weeks after discharge, a peak in physical activity was seen between 9-12 AM and at 5 PM.

**Conclusion:**

Older hospitalized patients spend most of their time being sedentary with their highest activity between 9-11 AM. Daily activity doubles after discharge with one extra peak in the afternoon. Daily routines might be disrupted, and older patients have the potential to be more physically active during hospitalization. Interventions that encourage physical activity during hospitalization are warranted.

**Supplementary Information:**

The online version contains supplementary material available at 10.1186/s12877-022-02917-8.

## Introduction

Lack of physical activity among patients aged 65 years or older is linked to several adverse health outcomes [1], such as chronic conditions, cardiovascular disease, diabetes [2], dementia [3], poor quality of life [4], cancer [2], rehospitalization [5] and mortality [6–9]**.** Worldwide, the proportion of older adults over 65 years of age is growing rapidly [10]. Similarly, hospitalization rates are expected to continue to increase [11, 12]. For instance, in 2018, persons aged 65 years and older accounted for more than 40% of Denmark's acute hospitalizations [13]. The increasing demand for healthcare in this segment of the population is a global phenomenon [12]. Consequently, attention is increasingly being given to factors contributing to more effective interventions and care for older (65+) patients during illness [14–17]. However, hospitalization is reported as an independent risk factor for loss of the ability to walk [18], loss of independence [19, 20], and functional decline after discharge, potentially leading to a higher level of sedentary behavior [18, 20, 21]. Multiple studies have assessed physical activity and sedentary behavior among older hospitalized patients [22] and have shown that older adults, including those who are able to walk independently [23–26], are inactive during the entire hospitalization period. The mean uptime (standing or walking) is reported to be 70 min. per day [22]. Also, a larger prospective study by Zisberg et al. 2007 [27] , assessing the effect of hospitalization-care processes in 330 hospitalized older adults age 70+, found clear disruptions to the self-reported frequency, duration, and timing of patients’ basic daily routines during hospitalization compared to pre-hospitalization. Preservation of basic daily routines is highly important, and promotes functional status, quality of sleep, and wellbeing [28–35]. Interestingly, Monk et al, assessed the lifestyle regularity in 100 healthy subjects with a mean age 31, and 104 seniors with mean age 79 years, found that irregularities in routines were linked to adverse outcomes such as depression, poor sleep quality and unhealthy aging [36, 37]. However, during hospitalization a hospital department’s routines may conflict with older patients’ routines. Therefore, an objective evaluation of the impact of hospitalization on patients’ activity patterns is needed. To the best of our knowledge, there are no previous longitudinal studies investigating physical activity and sedentary behavior with repeated objective measurement, focusing on pattern variation during hospitalization and after discharge. Hence, the aim of this study was to assess and compare daily and hourly patterns in step count, time spent in uptime and sedentary behavior during hospitalization and after discharge in a group of older patients (+65) using accelerometers.

## Methods

### Design and patients

This study is based on data from the randomized, controlled STAND-Cph trial, which recruited patients from September 2013 to September 2018 at Copenhagen University Hospital Hvidovre in Denmark and in the patients’ own homes. A full trial protocol is available with open access [38]. Briefly, the primary aim of the STAND-Cph trial was to investigate the effect of supervised, progressive strength training and post-training protein supplementation during and after hospitalization on mobility in older patients (≥ 65 years) admitted with acute medical illness. All included patients were home dwelling and were excluded on the following criteria: terminal illness; in treatment for diagnosed cancer; diagnosis of Chronic Obstructive Pulmonary Disease (COPD) and participation in a COPD rehabilitation program; inability to speak or understand Danish; inability to cooperate in tests/exercises; transfer to the intensive care unit; isolation-room stay; expected hospitalization lasting < 24 h; or inability to stand [38]. The patients were randomized to either the control group or the intervention group. This study involved patients allocated to the control group, who received routine care during hospitalization and following discharge [38]. The STAND-cph trail has been approved by the Ethics Committee of the Capital Region of Denmark (H-2-2012-115) and by the Danish Data Protection Agency (2007-58-0015). All participants gave written informed consent before participation, and the study was conducted in line with the Declaration of Helsinki.

### Assessment of physical activity

Physical activity in the form of hourly number of steps taken, time spent standing, walking, and sitting/lying (sedentary behavior) was assessed with an activity monitor (activPAL3™, PAL Technologies Ltd., Glasgow, UK). The patients were asked to wear the monitor at three time points: from the time of inclusion until discharge, for one week immediately after discharge, and for one week 4 weeks after discharge. An investigator attached the activPAL3™ to the patient´s right thigh halfway between the spina iliaca anterior superior and the patella. For the attachments and detachments after discharge, an investigator visited the patients in their own homes. The monitor was covered in Tegaderm™ transparent waterproof film (3 M, Maplewood, MN, USA) and attached to the patient by a PALstickie™ (dual-layer hydrogel adhesive pad). Hereafter, the activPAL3™ was covered by Leukomed® T transparent film (BNS medical, Hamburg, Germany) to enable the patients to wear the monitor while showering. The activPAL3™ was programmed to record continuously for 7 days at 20 Hz. Recording was started shortly before attachment to the patient and the start time and date were noted in a data log along with the time and date of attachment, non-wearing (reported by patient or clinical staff), and detachment of the monitor. After detachment, data were downloaded to a computer using the activPAL3™ Professional software version 7.2.32. We regarded a day to extend from 12:00 A.M. until 12:00 A.M. to optimize the number of full days with 24 h of measurement and to avoid half-day measurements, as patients were usually enrolled in the study in the morning. This was in accordance with a previous observational study prior to this randomized clinical trial [24]. To avoid the inclusion of distorted days in the analysis, only patient-days with more than 20 h of measurements were included [38]. Also, in the analysis we only included the first 6 days of hospitalization because very few patients were hospitalized for more than 6 days.

ActivPal3™ has been shown to be valid and reliable for measuring posture and purposeful walking in both young people and older adults [39, 40]. However, the monitor has limited reliability for measuring not purposeful walking and small steps movement. Validation of the first generation monitor ActivPAL reported less reliable data on walking speed of 0.45 m/s or lower [41, 42], which is likely the case for the ActivPal3™ as well since there is a good agreement between the first and second generation of monitors [43]. In a previous study from our hospital in 317 older medical patients, 46% walked at a speed below 0.67 m/s, and 34% at a speed below 0.56 m/s [44]. Therefore, as stated in our protocol paper [38], to account for possible inability of the monitor to distinguish between standing and walking at slow walking speed, time spent in walking and standing were combined into one category, uptime [45].

### Assessment of patient characteristics

After inclusion, baseline assessments were performed by an investigator. At discharge and four weeks after, the patients were reassessed in their own homes. The following descriptive variables were collected on admission: age, sex, weight (BMI), living status (marital status, type of residence, and living alone), co-morbidities, admission diagnose, history of smoking, use of ambulatory aids, use of municipal assistance and length of stay. The following were assessed at all assessments: Mobility by the New Mobility Score (NMS) (recall of mobility 2 weeks before admission and on the day of admission) [46] and the De Morton Mobility Index (DEMMI). The NMS is a self-report assessment of a person’s ability to perform indoor walking, outdoor walking and shopping and the level of assistance needed with a composite score of 0-9. The DEMMI is a valid and reliable mobility tool assessing bed mobility, chair mobility, static and dynamic balance, and walking [47–50]. The DEMMI is scored from 0 to 100 points with 100 points reflecting a high level of mobility and a score below 62 is considered limited mobility [51]; Activities of Daily Living (ADL) by the Bartel Index 20, which is scored between 0 and 20 with 20 reflecting no disability in ADL [52]; habitual gait speed (m/s) on a 4-m course [53]; cognitive impairment by the Short Orientation-Memory-Concentration test (OMC) [54], and habitual physical activity by a four-level self-reported questionnaire [55, 56].

### Data management and analysis

The collected data were double entered into Epidata Entry 3.1 by the first and last author and two assistants. Data from the activPAL3™ monitors were downloaded using activPAL™ Professional software version 7.2.32. The 15s Epoch files were used for analyses and were transferred to SAS Enterprise Guide 7.1 (SAS Institute Inc., Cary, NC, USA) and merged with data log information on wear time to assure that only wear time was included in the analyses. Data on time spent standing and walking were combined as time spent upright. From the 15s Epoch files both data on hourly and daily average were derived. Normally distributed data are presented as means with standard deviations and non-normally distributed data as medians with interquartile ranges. Categorical data are presented as frequencies with percentages. To determine changes in uptime (standing/walking), sedentary behavior (time spent lying/sitting) and steps, we used a mixed model using the SAS procedure PROC MIXED to calculate differences between hospitalization, discharge, and 4 weeks after discharge. When performing the analysis, we log transformed data for steps since these were only log-normally distributed. The chi-squared (χ2) test and the Student´s t test were used to determine differences between patients included in the analysis and those who dropped out with regards to sex, age and DEMMI-score.

## Results

### Baseline characteristics

In the STAND-Cph trial, 80 patients were randomized to the control group and thereby included in this study. The characteristics of the patients are shown in (Table 1). The patients´ median age was 80.9 years (IQR: 75;88), 68% were women, 98% were living at home, and 68% were living alone. At baseline, the median habitual walking speed was 0.67 (IQR: 0.48;0.87), the median DEMMI score was 57 (IQR: 48;67), and the median Bartel-20 score was 19 (IQR: 18;20). Thirty-one percent used assistive devices for walking and 37.5 % received social support services from the municipality. Prior to hospitalization, 46% of the patients were active two hours or more of per day based on self-report. The median length of stay was 4 days (IQR: 2;6.5), the median prevalence of co-morbidities was 4 (IQR: 3;5), and 41% were admitted to the hospital with respiratory symptoms.

**Table 1:** Baseline characteristics of patients.

Baseline variables	N	Overall
**Demographic characteristics**		
Age (median, IQR)	80	80.9 (75;88)
Sex (female, %)	80	55 (68%)
Length of stay (median, IQR)	80	4 (2;6.5)
BMI^1^ (median, IQR)	80	26 (22.6;30.1)
Comorbidities (median, IQR))	80	4 (3;5)
Living alone: (number, %)	80	54 (68%)
**Self-reported activity level prior to admission:**	**80**	
<2 hours (N, %)		43 (54%)
2-4 hours (N, %)		26 (33%)
>4 hour (N, %)		11 (13%)
**New mobility score** (median, IQR)		
Fourteen days prior to admission	80	7 (6;9)
Admission	80	6 (4;9)
DEMMI^2^ (median, IQR)	79	57 (48;67)
BARTEL-20 (median, IQR)	79	19 (18;20)
OMC ^3^ (median, IQR)	68	23 (18;26)
Walking speed m/s (median, IQR)	80	0.67(0.48;0.87)
**Smoking:**	80	64 (80%)
Smoking (no. yes, %)		13 (16.25)
Previous (no. yes, %)		51 (70.8%)
**Assistive device** (no., %)	80	26 (31%)
Walking stick		21 (26%)
Crutches		5 (6%)
**Use of municipal help**	80	30 (37.5%)
Personal assistance (no. yes, %)		7 (8.8%)
Food service (no. yes, %)		13 (16.3%)
Cleaning (no. yes, %)		10 (12.5%)
**Admission diagnosis**	80	
Pulmonary		33 (41%)
Cardiovascular		19 (24%)
Neurological		12 (15%)
Other		16 (20%)

*Results are expressed as median (interquartile range) for continuous variables and as number of participants (percentage) for categorical variables.* ^*1*^*: Body mass index (kg/m2).* ^*2*^*: De Morton Mobility Index.* ^*3*^*: Orientation-Memory-Concentration test.*

A total of 28 patients were lost to follow-up between hospitalization and 4 weeks after discharge. The reasons for missing data were: withdrawal from the study because of tiredness (n=10), withdrawal of consent (n=7), readmission with apoplexy (n=5), severe pain (n=3), cancer (n=1), abdominal surgery (n=1), and loss of contact with the subject (n=1). There were no significant baseline differences in age, sex, Barthel-score or DEMMI-score between patients lost to follow-up and patients remaining in the study.

### Variation in steps, uptime and sedentary behavior measured during hospitalization, after discharge and after 4 weeks.

Sixty-five patients wore the accelerometer at one or more assessment timepoints. In total, 48 patients wore the accelerometer during hospitalization, 49 after discharge, and 43 patients four weeks after discharge. The distribution of collected activPAL3 data from the three assessment timepoints is summarized in Supplementary 1.

In total, 21.216 h of patient activity were recoded (5710 h during hospitalization, 8232 h after discharge and 7224 h four weeks after discharge). The median number of steps taken per day was 728 (IQR: 176;2089) during hospitalization, 2207 (IQR: 1433;3148) after discharge and 2622 (IQR:1714;3864) four weeks after discharge. The median time per day spent in uptime was 1.7h (IQR:1.0;2.8) during hospitalization, 4.0 h (IQR: 2.7;5.4) after discharge, and 4.0 h (IQR: 2.8;5.8) four weeks after discharge. For sedentary behavior the median time per day was 21.4 h (IQR: 20.7;22.4) during hospitalization, 19.5 h (IQR: 18.1;21.0) after discharge and 19.6 h (IQR: 18.0;20.8) four weeks after discharge. After discharge, the patients’ number of steps and uptime significantly increased and sedentary behavior significantly decreased compared to hospitalization (p<0.0001). the steps number increased by 204 % [149;271%], the mean uptime increased with 1.87 h [1.5;2.24] and the mean sedentary time decreased with 1.89 h [- 2.28; -1.50]. Overall, also a significant increase was seen between discharge and four weeks after discharge (p<0.01). The number of steps increased by 28 % [10 %; 50 %], uptime increased with 0.57 h [0.28;0.87], and sedentary time decreased with 0.48 h [-0.79; -0,67]. When looking at the patients’ DEMMI score, those with limited mobility (DEMMI ≤ 62 had a relatively higher increase in activity between hospitalization and discharge compared to those with non-limited mobility (DEMMI > 62) (242 % versus 172 %, Table 2). Also, an increase between discharge and 4 weeks after discharge was only seen in those with a DEMMI >62 (Table 2).

**Table 2:** Number of steps, time spent in uptime and sedentary behavior during the three periods.

Test time:	Patients (N)	Steps (number per day)	Uptime (hours per day)	Sedentary behavior (hours per day)
Hospitalization	48	728 (IQR: 176;2089)	1.7 h (IQR: 1.0;2.8)	21.4 h (IQR: 20.7;22.4)
Discharge	49	2207 (IQR: 1433;3148)	4.0 h (IQR: 2.7;5.4)	19.5 h (IQR: 18.1;21.0)
Four weeks after discharge	43	2622(IQR: 1714;3865)	4.0 h (IQR: 2.8;5.8)	19.6 h (IQR: 18.0;20.8)
Change from hospitalization to discharge		204 % [CI: 149 ;271%] *	1.87 h [CI: 1.5;2.24] *	-1.89 h [CI: - 2.28; -1.50] *
*DEMMI > 62*	28	172 % [CI: 108; 257 %] *	1.02 h [CI: 0.83;1.22] *	-1,23 h [CI: -1.80; -0.67] *
*DEMMI ≤ 62*	37	242 % [CI: 157; 353 %] *	2.86 h [CI: 2-43;3.29] *	-3.10 h [CI: -3.60; -2,58] *
Change from discharge to four weeks after		28 % [CI: 10 %; 50 %] **	0.57 h [CI: 0.28;0.87] **	-0.48 h [CI: -0.79; -0,67] **
DEMMI > 62	28	62 % [CI: 34; 96 %] **	0.23 h [CI: 0.09;0.37] **	-0.69 h [CI: -1.11; -0.27] **
DEMMI ≤ 62	37	-2 % [CI: -24; 26 %]	0.28 h [CI: -0.08;0.65]	-0.09 h [CI: -0.50; -0.33] **

***Table*** ***2***: Results for daily number of steps, time spent in uptime and sedentary behavior during hospitalization, after discharge and four weeks after discharge. The results for taken number of steps and time (h) spent in uptime/sedentary are expressed as medians with first and third quartile. Changes between test times are expressed as means with 95% Confidence interval (CI). relative changes are expressed as percentage for steps and absolute changes for uptime and sedentary behavior; * p<0.0001 for the difference between baseline (hospitalization) and discharge;** p<0.01 for the difference between discharge (baseline) and four weeks after discharge.

### Hourly variation

**Fig. 1:**
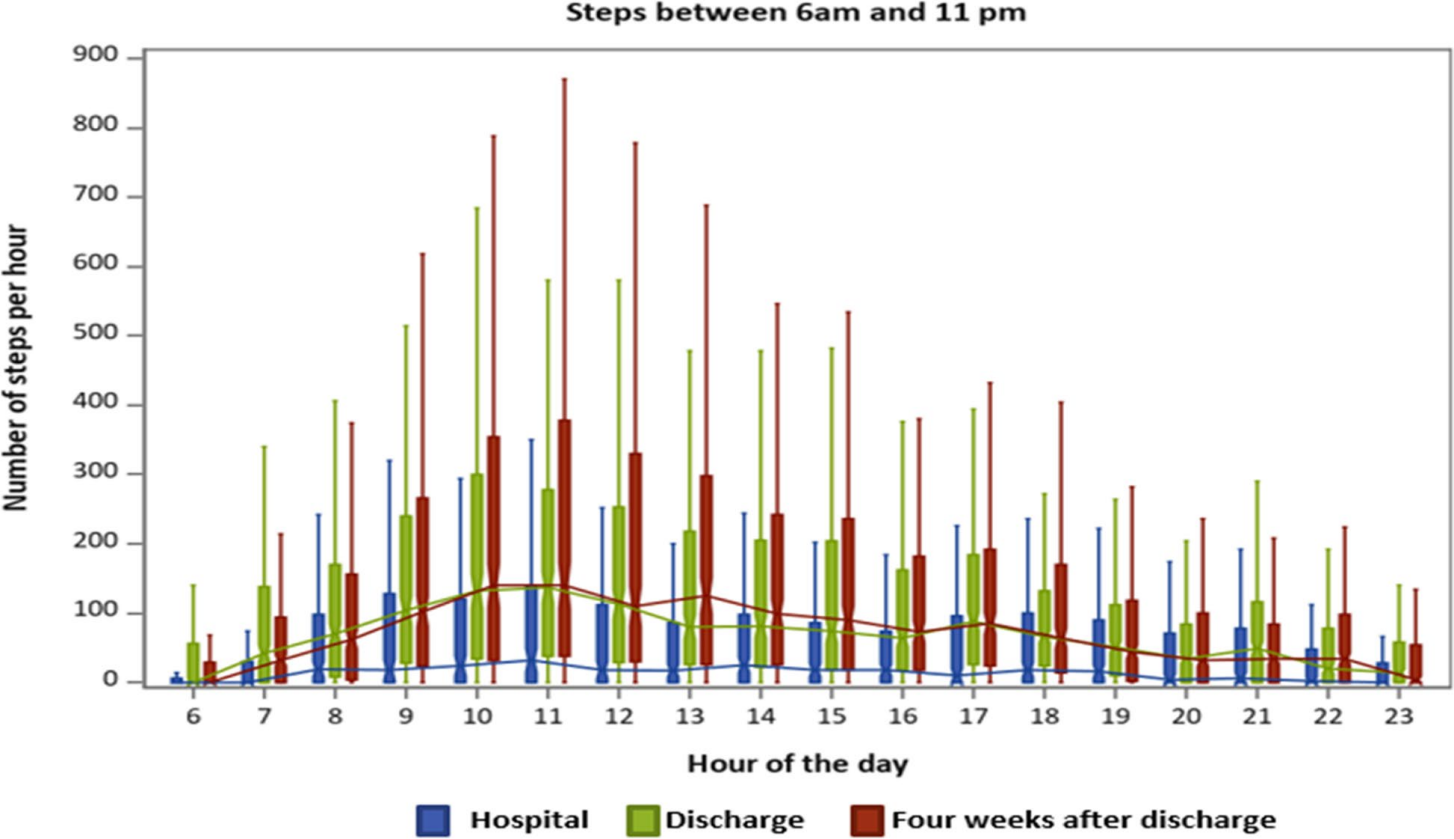
Number of steps taken per hour from 6 am to 11 pm. Boxplots illustrating lower quartile, median, upper quartile and extremes per hour during hospitalization (blue), after discharge (green) and four weeks after discharge (red). The lines (blue, red, green) connect the medians during the day

**Fig. 2:**
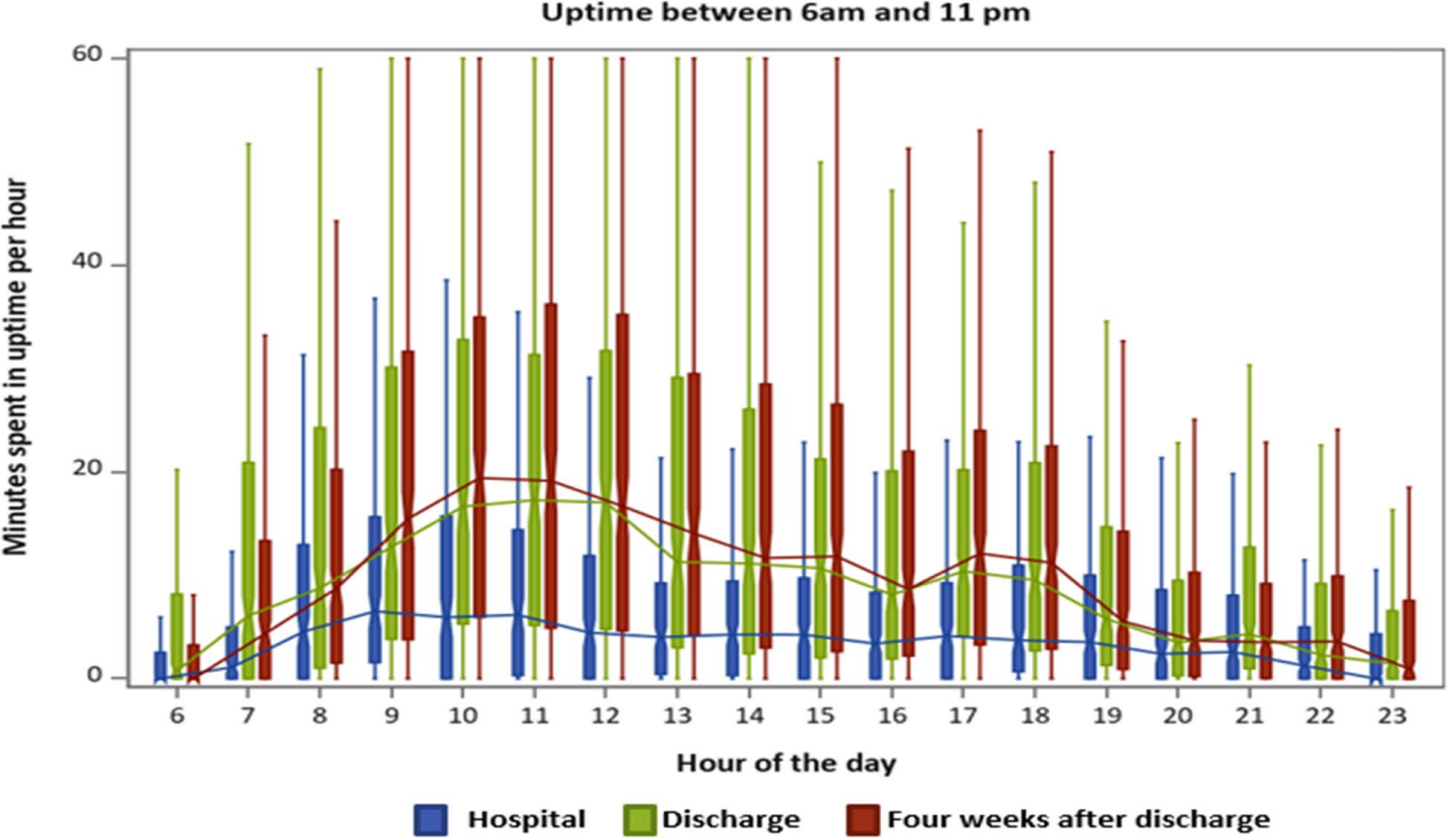
Time spent standing or walking (uptime) in minutes per hour from 6 am to 11 pm. Boxplots illustrating lower quartile, median, upper quartile and extremes in minutes per hour during hospitalization (blue), after discharge (green) and four weeks after discharge (red). The lines connect the medians during the day

**Fig. 3:**
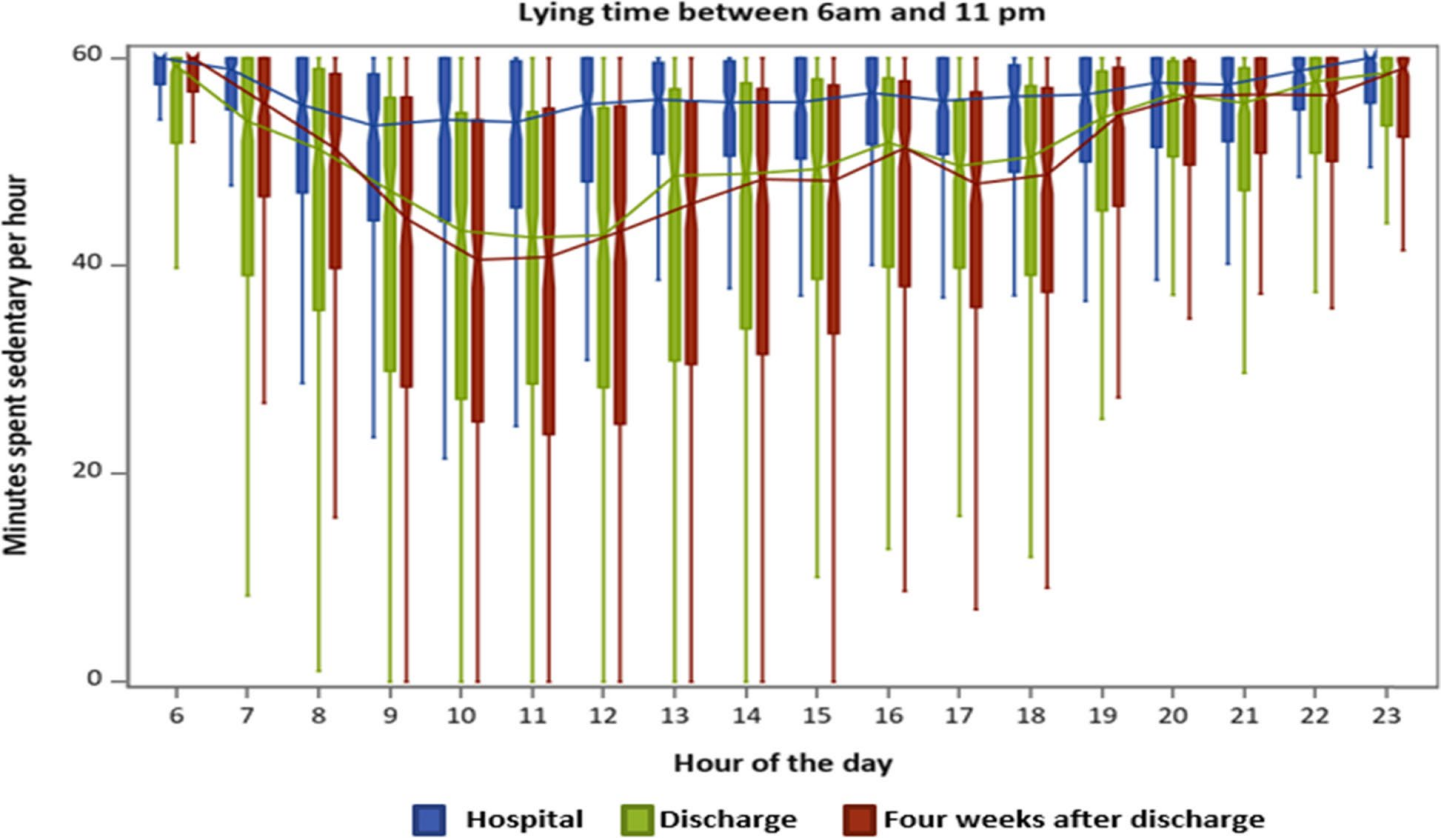
Time spent sitting or lying (sedentary behavior) in minutes per hour from 6 am to 11 pm. Boxplots with lower quartile, median, upper quartile and extremes in minutes per hour during hospitalization (blue), after discharge (green) and four weeks after discharge (red). The lines connect the medians during the day

The hourly variations in steps and uptime and sedentary behavior are presented in Figures 1,2 and 3, respectively. The figures show the activity distribution within eighteen hours of monitoring (nighttime not included in the figures). During hospitalization, no notable variation in steps, uptime and sedentary behavior per hour was seen after 9 AM apart from a small two-hour peak in steps and uptime (Figures 1 and 2) and decline in sedentary time (Figure 3) at 10 - 11 AM. After discharge and 4 weeks after discharge, the patients' diurnal profiles changed and the patients took more steps at all hours and spent more time in uptime than during hospitalization with most activity occurring between 9 and 12 AM and with an additional activity peak at 5-6 PM. During hospitalization, between 10–11 AM, when the patients' exhibited the least sedentary behavior, the median time in minutes per hour for sedentary behavior was 53.5 minutes (IQR: 44.3;60). After discharge and four weeks after discharge, between 10 – 11 AM, when the patients' exhibited the least sedentary behavior the patients’ median time spent in sedentary behavior was 43 minutes (IQR: 28.2;55.1).

## Discussion

In this study, physical activity and the diurnal profile of activity during and up to one month after hospitalization was investigated with an accelerometer in a group of older patients hospitalized for acute illness. During hospitalization, the patients spent more time engaged in sedentary behavior and took fewer steps and were less physically active than after discharge. The included patients took less than 900 steps per day and were therefore at risk of hospitalization-associated functional decline [16]. Also, during hospitalization the diurnal profiles for steps and uptime had no notable variation in activity after 9 AM. In contrast, we found a diurnal profile for steps and uptime after discharge and four weeks after discharge with most physical activity occurring between 9 AM-12 PM, and 5-6 PM and with the patients being more physically active throughout the day than during hospitalization.

### Levels of activity

During hospitalization, the patients spent a median of 21.4 h of the day engaged in sedentary behavior and spent a median of 1.7 hours upright. This is consistent with a review of studies in acute-care settings in which the daily time spent lying or sitting accounted for 89–99%, and the duration of uptime was 1–2 hours per day [22]. After discharge a doubling in uptime was seen. Our findings are consistent with a recent study by Kolk et al. [57], which aimed to measure the number of steps taken per day in hospital and up to one week after discharge in 188 old (+70) acutely hospitalized patients, with Fitbit Flex worn on the wrist. In contrast, their study did not measure the distribution of physical activity and sedentary through the day. However, comparable findings were seen. Kolk et al. showed a doubling in steps only one day after discharge compared with one day prior to discharge - from 945 steps (IQR: 367;1943) to 1750 (IQR:675;4114). Presumably, the relatively high level of activity after discharge in both our study and the study by Kolk et al. indicate a recovery of activity the first week after discharge. This doubling in the duration of uptime, and the consequent decrease in sedentary time after discharge, suggests that sedentary behavior is a result of a culture of bed rest at the departments and that the patients could potentially be more physical active. This is well in line with recent studies from our department. In an ethnographic study with observations of daily practice in the departments [58], mobility of older medical patients was found to be dependent on the health professionals’ different cultural models, which ended up blurring the responsibility for ensuring patient mobility and ended up restricting patient mobility. Also, Pedersen et al. 2020 conducted a qualitative study that investigated facilitators and barriers for mobility during hospitalization, by semi-structed interviews with twelve physicians at two medical departments and Stefánsdóttir et al. explored 20 old (+65) medical patients’ experiences with mobility during hospitalization more generally, and with an intervention to increase in-hospital mobility. Pedersen et al. 2020 [59] and Stefánsdóttir et al. [60] found that barriers for mobility in older medical patients were the provision of excessive service and care by the department, a culture of bed rest, and lack of encouragement by health care professionals to motivate the patients to increase activity. Also, Stefansdottir et al. reported that the staff brought food, beverages, and clothes to patients, including those who were able to get out of bed and walk.

### Diurnal profiles

A second important finding was that during hospitalization only one minor peak in uptime occurred at 9 AM. This suggests that the patients only get out of the bed in the morning, maybe for the morning toileting or breakfast, and spent most time engaged in sedentary behavior for the rest of the day. However, right after discharge and four weeks later, two peaks in activity occurred at similar timepoints throughout the day. A study from Germany, by Mai et al. [61] analyzed the diurnal physical activity profile in 149 non-hospitalized, chronically ill community-dwelling individuals older than 70 years. The participants were instructed to wear a pedometer on six consecutive days. Consistent with our study, they identified two peaks. However, their timing for the peaks was advanced one-two hours compared with our results, one at 10-11 AM and a second at 3-4 PM. They found sex, age, morbidity, and season to have no moderating effects and that limited mobility was the only factor that significantly moderated the profile, reducing the number of peaks to one [61]. In our sample, the median DEMMI-score was 57 (reflecting limited mobility) on admission. Thus, limited mobility can also be the reason for the lack of a second peak of activity in our group during hospitalization since the median DEMMI score in the included patients was 57 on admission, which reflect the patients are limited in their mobility and have increased reliance on care and caregivers [51]. In contrast to our study, a recent study from Switzerland by Tasheva et al. [62], found three peaks of physical activity during the day: between 8-10 AM, at 12 PM, and at 6 PM. Tasheva et al. assessed the distribution of physical activity levels continuously during the hospital stay by a wrist accelerometer in 177 old (+65) patients hospitalized for acute medical illness. The authors proposed that older inpatients are primarily active during meals, as reflected in the three peak times. Although the patients in our study did receive three meals per day, this was not reflected in their peak times and may indicate that meals were consumed close to or in bed, and that the patients in the study by Tasheva et al. had the possibility to consume their meals in e.g. a dining room at the hospital. Moreover, we consider the above-mentioned barriers to be explanations for the lack of a second peak during hospitalization in our study. The diurnal profiles of the patients after discharge and four weeks after discharge show that the patients started physical activity one hour earlier and had higher levels of physical activity in the morning and afternoon hours. These findings are consistent with those of Zisberg et al. [28], who showed that the timing of getting dressed in the morning moved an hour and a half during hospitalization, and most basic activities were reduced in frequency and duration.

### Daily routines

Our findings indicate that older patients could potentially be more physically active during hospitalization and emphasize the need for interventions that encourage more physical activity during hospitalization. It could be in the form of simple routine activities, such as patients eating their breakfast out of bed in a common room and changing clothes by themselves. Another starting point for the effort to increase patients’ physical activity during hospitalization could be to encourage physical activity around 10-12 AM, and 5-6 PM, which would be more consistent with their habitual behavior. It is known that older patients are vulnerable to disturbances in their routines [63]. Thus, suggested efforts should be made to re-establish routines among those at risk of loss of functional decline. Since our results showed a wide range in steps, uptime and sedentary behavior, a personalized intervention strategy would be a reasonable means of optimizing physical activity during hospitalization. Recently, guidelines on physical activity for admitted older patients have also highlighted the importance of integrating physical activity throughout daily care, with a focus on functionality and activities of daily living; and bearing in mind that it is important for patients and staff to share the responsibility of promoting physical activity and minimizing sedentary behavior [15]. An alternative method for providing clinical care to a segment of this group of older patients has recently been suggested in a systematic review [64], which found that hospital-at-home (HaH) treatment may be a clinically effective approach and suggested that this treatment method may result in less functional decline in patients than the traditional ward-based treatment method. However, further research is needed, and the implementation of this alternative method of treatment (HaH) would necessitate significant changes to the current practice as well as time, it can takes several years making structural changes in the healthcare systems. In the short term, a relevant indicator is needed to identify patients at a high risk of inactivity during their time in the hospital. In the long term, systematic changes in the hospital environment and care setting are needed where the responsibility to encourage physical activity should be a shared responsibility and delegated to all health professionals as suggested in recent recommendations for physical activity [15].

## Strengths and limitations

This study’s major strength was the longitudinal measurement of physical activity during hospitalization, at discharge, and at four weeks after discharge in a heterogeneous cohort of older adults hospitalized for acute illness. This study has some limitations. First, physical activity was not recorded prior to hospitalization. An objective assessment of physical activity prior to hospitalization would give a more sufficient picture of the impact of hospitalization on physical activity. However, this would require assessments of a broad range of older adults to ensure that some of those who are hospitalized were assessed prior to hospitalization. Therefore, self-report on pre-hospitalization activity is easier to collect. Secondly, we assessed the number of steps using the activPAL3™ activity monitor. The first generation of activPAL had an uncertainty in detecting walking at speeds less than 0.45 m/s [40, 41] and not purposeful walking. It is uncertain if the activPAL3™ has the same issues, however a study has reported a similarity between the two generations of activPAL accelerometers [43]. At baseline, the patients in the current study had a median walking speed of 0.67m/s (IQR: 0.48;0.87), and it is therefore likely that walking and steps were underestimated in the patients who had a gait speed below the first quartile. However, we considered that an underestimation would affect the absolute level of steps, but not affect the distribution of activity throughout the day, which is one of the reasons why we looked at uptime (walking and standing), we therefore believe that the diurnal physical activity profiles are a true reflection of the included older adults' patterns of daily physical activity.

## Conclusion

This study showed that in older acutely admitted adults the diurnal activity profile during hospitalization was distinct from the diurnal profile when the patients returned home. During hospitalization, the patients took fewer steps, spent less time standing and walking, and spent more of their time engaged in sedentary behavior. The first week after discharge, the patients doubled their time spent standing and walking and lowered their time spent in sedentary behavior, suggesting that sedentary behavior is a result of a culture of bed rest in the hospital. Therefore, general mobility regimes and motivation should be provided to all patients during acute hospitalization.

## Supplementary Information

**ESM 1.**

## Data Availability

The data supporting the conclusions of this article are included within the article. The datasets analysed in this study are not publicly available due to regulations set out by the European and Danish Data Protection Agency regarding data anonymization but are available from the corresponding author on reasonable request.

## Abbreviation

IQR: interquartile range
BMI: body mass index
DEMMI: De Morton Mobility Index
MSE: Mini-Mental State Examination
NMS: New mobility score

## References

[1] Musich S (2017). The Frequency and Health Benefits of Physical Activity for Older Adults. Popul Health Manag.

[2] O., World Health (2018). Global action plan on physical activity 2018–2030: more active people for a healthier world.

[3] Livingston G (2017). Dementia prevention, intervention, and care. Lancet.

[4] Camboim FENDF (2017). Benefits of physical activity in the third age for the quality of life. Journal of Nursing Ufpe. Online.

[5] Fisher SR (2016). Inpatient Walking Activity to Predict Readmission in Older Adults. Archives of physical medicine and rehabilitation.

[6] Ostir GV (2013). Mobility activity and its value as a prognostic indicator of survival in hospitalized older adults. J Am Geriatr Soc.

[7] Buchman AS (2012). Total daily physical activity and longevity in old age. Arch Intern Med.

[8] Lee, I.M., et al., Association of Step Volume and Intensity With All-Cause Mortality in Older Women. JAMA Intern Med, 2019. 10.1001/jamainternmed.2019.0899 PMC6547157 31141585

[9] Hupin D (2015). Even a low-dose of moderate-to-vigorous physical activity reduces mortality by 22% in adults aged ≥60 years: a systematic review and meta-analysis. Br J Sports Med.

[10] United Nations, D.o.E.a.S.A., World Population Prospects: The 2015 Revision, Key Findings and Advance Tables. 2015.

[11] Wu, X., C.-k. Law, and P.S. Yip, A Projection of Future Hospitalisation Needs in a Rapidly Ageing Society: A Hong Kong Experience. International Journal of Environmental Research and Public Health, 2019. **16**(3). 10.3390/ijerph16030473 PMC6388233 30736289

[12] de Meijer C (2013). The effect of population aging on health expenditure growth: a critical review. Eur J Ageing.

[13] 2018, S.D., Statistics Denmark 2018, INDO1: admission by region, diagnosis,age and sex, viewed 13. December 2020, < https://www.statistikbanken.dk/IND01. 2018.

[14] Krumholz HM (2013). Post-hospital syndrome--an acquired, transient condition of generalized risk. N Engl J Med.

[15] Baldwin CE (2020). Recommendations for older adults' physical activity and sedentary behaviour during hospitalisation for an acute medical illness: an international Delphi study. Int J Behav Nutr Phys Act.

[16] Agmon M (2017). Association Between 900 Steps a Day and Functional Decline in Older Hospitalized Patients. JAMA Intern Med.

[17] Cortes OL, Delgado S, Esparza M (2019). Systematic review and meta-analysis of experimental studies: In-hospital mobilization for patients admitted for medical treatment. J Adv Nurs.

[18] Mahoney JE, Sager MA, Jalaluddin M (1998). New walking dependence associated with hospitalization for acute medical illness: incidence and significance. J Gerontol A Biol Sci Med Sci.

[19] Covinsky KE (2003). Loss of independence in activities of daily living in older adults hospitalized with medical illnesses: increased vulnerability with age. J Am Geriatr Soc.

[20] Dharmarajan K (2020). Disability and Recovery After Hospitalization for Medical Illness Among Community-Living Older Persons: A Prospective Cohort Study. J Am Geriatr Soc.

[21] Zisberg A (2015). Hospital-associated functional decline: the role of hospitalization processes beyond individual risk factors. J Am Geriatr Soc.

[22] Fazio S (2020). How much do hospitalized adults move? A systematic review and meta-analysis. Appl Nurs Res.

[23] Brown CJ (2009). The underrecognized epidemic of low mobility during hospitalization of older adults. J Am Geriatr Soc.

[24] Pedersen MM (2013). Twenty-four-hour mobility during acute hospitalization in older medical patients. J Gerontol A Biol Sci Med Sci.

[25] Villumsen M (2015). Very Low Levels of Physical Activity in Older Patients During Hospitalization at an Acute Geriatric Ward: A Prospective Cohort Study. J Aging Phys Act.

[26] Fisher SR (2011). Ambulatory activity of older adults hospitalized with acute medical illness. Journal of the American Geriatrics Society.

[27] Zisberg A, Gur-Yaish N (2017). Older adults' personal routine at time of hospitalization. Geriatr Nurs.

[28] O'Conor R (2019). Daily Routine: Associations With Health Status and Urgent Health Care Utilization Among Older Adults. Gerontologist.

[29] Clark, F., et al., Occupational therapy for independent-living older adults. A randomized controlled trial. JAMA, 1997. **278**(16): p. 1321-6. 9343462

[30] Clark F (2001). Embedding health-promoting changes into the daily lives of independent-living older adults: long-term follow-up of occupational therapy intervention. J Gerontol B Psychol Sci Soc Sci.

[31] Foldvari M (2000). Association of muscle power with functional status in community-dwelling elderly women. J Gerontol A Biol Sci Med Sci.

[32] Resnick B, Galik E, Boltz M (2013). Function focused care approaches: literature review of progress and future possibilities. J Am Med Dir Assoc.

[33] Resnick B (2016). Dissemination and Implementation of Function Focused Care for Assisted Living. Health Educ Behav.

[34] Zisberg A, Gur-Yaish N, Shochat T (2010). Contribution of routine to sleep quality in community elderly. Sleep.

[35] Ludwig FM (1997). How routine facilitates wellbeing in older women. Occupational Therapy International.

[36] Monk TH (2006). Age-related differences in the lifestyle regularity of seniors experiencing bereavement, care-giving, insomnia, and advancement into old-old age. Chronobiol Int.

[37] Monk TH (2003). The relationship between lifestyle regularity and subjective sleep quality. Chronobiol Int.

[38] Pedersen MM (2016). Supervised progressive cross-continuum strength training compared with usual care in older medical patients: study protocol for a randomized controlled trial (the STAND-Cph trial). Trials.

[39] Bourke A, Ihlen E, Helbostad J. Validation of the activPAL in Free-Living and Laboratory Scenarios for the Measurement of Physical Activity. Stepping, and Transitions in Older Adults. 2019:1–8.

[40] Sellers C, et al. Validity and reliability of the activPAL3 for measuring posture and stepping in adults and young people. Gait & Posture. 2015;**43**. 10.1016/j.gaitpost.2015.10.020 26669950

[41] Taraldsen K (2011). Evaluation of a body-worn sensor system to measure physical activity in older people with impaired function. Phys Ther.

[42] Kanoun N. Validation of the ActivPAL Activity Monitor as a Measure of Walking at Pre-determined Slow Walking Speeds in a Healthy Population in a Controlled Setting'. Reinvention. Journal of Undergraduate Research. 2009;**2**(2).

[43] Sellers C, et al. Agreement of the activPAL3 and activPAL for characterising posture and stepping in adults and children. Gait & Posture. 2016;**48**. 10.1016/j.gaitpost.2016.05.012 27318305

[44] Bodilsen AC (2016). Prediction of Mobility Limitations after Hospitalization in Older Medical Patients by Simple Measures of Physical Performance Obtained at Admission to the Emergency Department. PLoS One.

[45] Pedersen, M.M., et al., A randomized controlled trial of the effect of supervised progressive cross-continuum strength training and protein supplementation in older medical patients: the STAND-Cph trial. Trials, 2019. **20**(1): p. 655-655. 10.1186/s13063-019-3720-x PMC6883554 31779693

[46] Kristensen MT, Foss NB, Kehlet H (2005). Timed Up and Go and New Mobility Score as predictors of function six months after hip fracture. Ugeskr Laeger.

[47] de Morton NA, Davidson M, Keating JL (2008). The de Morton Mobility Index (DEMMI): an essential health index for an ageing world. Health Qual Life Outcomes.

[48] Davenport SJ, de Morton NA (2011). Clinimetric properties of the de Morton Mobility Index in healthy, community-dwelling older adults. Arch Phys Med Rehabil.

[49] de Morton NA, Lane K (2010). Validity and reliability of the de Morton Mobility Index in the subacute hospital setting in a geriatric evaluation and management population. J Rehabil Med.

[50] de Morton NA, Davidson M, Keating JL (2010). Validity, responsiveness and the minimal clinically important difference for the de Morton Mobility Index (DEMMI) in an older acute medical population. BMC Geriatr.

[51] Macri EM (2012). The de morton mobility index: normative data for a clinically useful mobility instrument. J Aging Res.

[52] Collin C (1988). The Barthel ADL Index: a reliability study. Int Disabil Stud.

[53] Guralnik JM (2000). Lower extremity function and subsequent disability: consistency across studies, predictive models, and value of gait speed alone compared with the short physical performance battery. J Gerontol A Biol Sci Med Sci.

[54] Katzman R (1983). Validation of a short Orientation-Memory-Concentration Test of cognitive impairment. Am J Psychiatry.

[55] Schnohr P, Scharling H, Jensen JS (2003). Changes in leisure-time physical activity and risk of death: an observational study of 7,000 men and women. Am J Epidemiol.

[56] Saltin B, Grimby G (1968). Physiological analysis of middle-aged and old former athletes. Comparison with still active athletes of the same ages. Circulation.

[57] Kolk D (2021). Factors Associated with Step Numbers in Acutely Hospitalized Older Adults: The Hospital-Activities of Daily Living Study. J Am Med Dir Assoc.

[58] Kirk JW (2019). Disentangling the complexity of mobility of older medical patients in routine practice: An ethnographic study in Denmark. PLoS One.

[59] Pedersen MM (2020). Is Promotion of Mobility in Older Patients Hospitalized for Medical Illness a Physician's Job?-An Interview Study with Physicians in Denmark. Geriatrics (Basel, Switzerland).

[60] Stefánsdóttir N (2020). Older medical patients' experiences with mobility during hospitalization and the WALK-Copenhagen (WALK-Cph) intervention: A qualitative study in Denmark. Geriatr Nurs.

[61] Mai A (2014). Diurnal profiles of pedometer-determined physical activity in chronically ill and mobility-limited older adults: a cross-sectional study. BMC Public Health.

[62] Tasheva P (2020). Accelerometry assessed physical activity of older adults hospitalized with acute medical illness - an observational study. BMC Geriatr.

[63] Stewart NH, Arora VM (2018). Sleep in Hospitalized Older Adults. Sleep medicine clinics.

[64] Scott J, et al. A systematic review of the physical activity levels of acutely ill older adults in Hospital At Home settings: an under-researched field. Eur Geriatr Med. 2020:1–12. 10.1007/s41999-020-00414-y PMC7557152 33058019

