# Supplementary material for: Variations in physical activity and sedentary behavior during and after hospitalization in acutely admitted older medical patients: a longitudinal study
Source: BMC Geriatr. 2022 Mar 15;22:209. doi: 10.1186/s12877-022-02917-8 (PMC8925078; doi:10.1186/s12877-022-02917-8)
Supplement: Supplementary file 1 — ESM 1. [file 12877_2022_2917_MOESM1_ESM.docx]

Supplementary Table 1:

**DISTRIBUTION OF INCLUDED ACTIVPAL3 DATA FROM THE TREE ASSESMEMT TIMEPOINTS**

Supplementary Table 1: + = included participant data, - = missing data and not included. The table shows the distribution of included ACTIVEPAL3 data from the tree assessments timepoints. 20% of the data comes from patients who only wore the activePAL3 during hospitalization, 11% comes from patients who wore activPAL3 during hospitalization and after discharge, 40% wore activPAL3 in alle tree assessments timepoints and 21,5% comes from patients wore only wore ACTIVPAL3 during the last to assessment: after discharge and four weeks after discharge.

| **Hospitalization**  **(Included data)** | **Discharge**  **(included data)** | **4 weeks after discharge**  **(included data)** | **Frequency of each configuration** | **Frequency of each configuration (%)** |
| --- | --- | --- | --- | --- |
| + | - | - | 13 | 20 % |
| + | + | - | 7 | 11 % |
| + | + | + | 26 | 40 % |
| - | + | + | 14 | 21.5 % |
| - | - | + | 1 | 1.5 % |
| - | + | - | 2 | 3 % |
| - | - | + | 2 | 3 % |
| **Total = 48** | **Total = 49** | **Total = 43** | **Total = 65** | **Total = 100%** |
